# Back Pain Due to Kummell’s Disease

**DOI:** 10.7759/cureus.18355

**Published:** 2021-09-28

**Authors:** Gurubharath Ilangovan, Narmada DA, Nikhil Murugadass, Zoubir Boudi, Shamaila Masood-Husain, Akshaya S Bhagavathula, Pooja Varwatte, Moien AB Khan

**Affiliations:** 1 Radiology, Chettinad Hospital and Research Institute, Chettinad Academy of Research and Education, Chennai, IND; 2 Emergency Medicine, Dr Sulaiman Al Habib Hospital, Dubai, ARE; 3 Family Medicine, Haling Park Medical Practice, London, GBR; 4 Public Health, Institute of Public Health, College of Medicine and Health Sciences, United Arab Emirates University, Al Ain, ARE; 5 Radiology, Shri Sathya Sai Medical Hospital and Research Institute, Chennai, IND; 6 Family Medicine, College of Medicine and Health Sciences, United Arab Emirates University, Al Ain, ARE; 7 Primary Care, North West London - National Health Service Provider, London, GBR

**Keywords:** preventive orthopedics, intervertebral fluid cleft, vertebral body, back pain, vertebral fracture, avascular osteonecrosis, kummell’s disease

## Abstract

Kummell’s disease (KD) is a delayed post-traumatic avascular osteonecrosis of the vertebral body secondary to a vertebral compression fracture that can present with back pain. We discuss the importance of an accurate diagnosis and appropriate management of Kummell’s disease. Additionally, we aim to increase awareness and promote early diagnosis and treatment to prevent serious complications.

A 55-year-old man had been diagnosed with avascular necrosis (AVN) of both hips and had a history of trauma to the left hip ten years ago. Between the initial fall and subsequent presentation, he resumed independent physical activity. At approximately 10 months following his initial injury, he returned to a local emergency department with vague complaints of lower back pain. He was prescribed analgesics for pain and discharged. Subsequently, he experienced a progressive increase in pain and complained of motor deficits of the lower limbs. He presented to our emergency room with acute onset of worsening pain. Magnetic resonance imaging revealed multiple-level compression fractures and a fluid cleft in the L2 intervertebral disc. Surgery was advised, but he did not consent. Therefore, nonsurgical treatment included bed rest, wearing a brace, lumbar traction, analgesics, and medication to prevent osteoporosis.

Prompt, accurate diagnosis of Kummell’s disease is important for timely, appropriate treatment, which can improve quality of life and prevent comorbidities.

## Introduction

Avascular necrosis (AVN), Kummel’s disease (KD), or necrosis of the vertebral body was described by the German surgeon Hermann Kümmell who reported the case history of six patients in 1895. He defined it as a vertebral body collapse occurring long after a minor spinal traumatic event that follows an initial asymptomatic phase followed by a symptomatic period of pain and angular kyphosis [1]. Several pathogenetic mechanisms underlying KD have been proposed including atrophic non-union, microfracture, fatigue fracture, pseudarthrosis, and AVN, secondary to vertebral body collapse [1]. Serial radiographic imaging is necessary to identify the worsening symptoms and diagnose the disease. However, there have been no reports of radiographical confirmation of a patient with posttraumatic avascular osteonecrosis and a fluid cleft in the L2 vertebral body. Here, we describe a KD patient with vertebral osteonecrosis and a fluid cleft in the L2 vertebral body and discuss the radiographic findings.

## Case presentation

A 55-year-old man presented to the emergency room complaining of lower back pain. He had a one-week history of pain in the left hip associated with difficulty in walking. He reported acute pain from the onset that progressed within a few days. The initial trauma to the left hip occurred ten years prior and caused AVN of both hips. He received native medical treatment at that time and was deferred for surgery. No other comorbidities or radiographic evidence of previous trauma was obtained during the visit.

The patient reported successful mobilization and discharge following therapy after the initial trauma, and since then, he had resumed daily activities. At approximately ten months following the initial injury, he returned to a local emergency department with vague complaints of lower back pain. After an unremarkable workup, he was prescribed analgesics and discharged.

At the presentation to our emergency department, his back pain was progressively increasing, and motor deficits in the lower limbs accompanied the acute onset of worsening pain. The patient appeared moderately built and nourished. The physical examination revealed apparent shortening of the left hip and pain during a range of movements. Distal pulses were found in both lower limbs.

**Figure 1:**
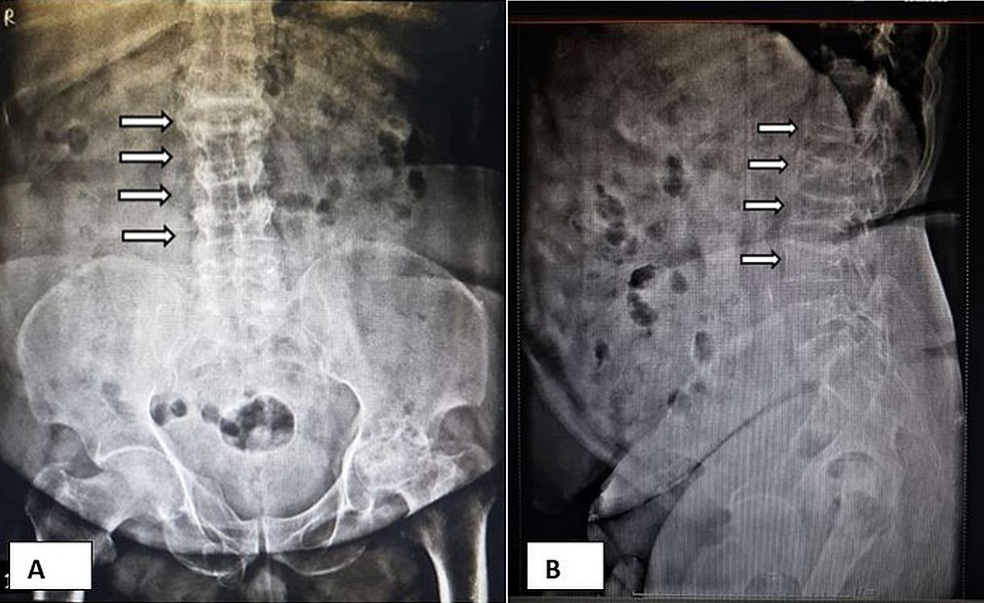
(A) Anteroposterior view of the lumbosacral spine and (B) lateral view of the lumbosacral spine Lumbosacral spine shows loss of normal lumbar curvature and collapse (arrows) of multiple vertebral bodies (T12, L1, L2, and L3), with a reduction in vertebral body height and a reduction in the left acetabular femoral joint space with advanced degenerative changes in the left hip joint. T: thoracic; L: lumbar.

A lumbosacral spinal radiograph (lateral view) revealed loss of normal lumbar curvature and collapse of multiple vertebral bodies accompanied by a reduction in vertebral body height. Degenerative changes in the left hip joint along with a reduction in the left acetabular femoral joint space were also observed on the X-ray (Figure 1A).

**Figure 2:**
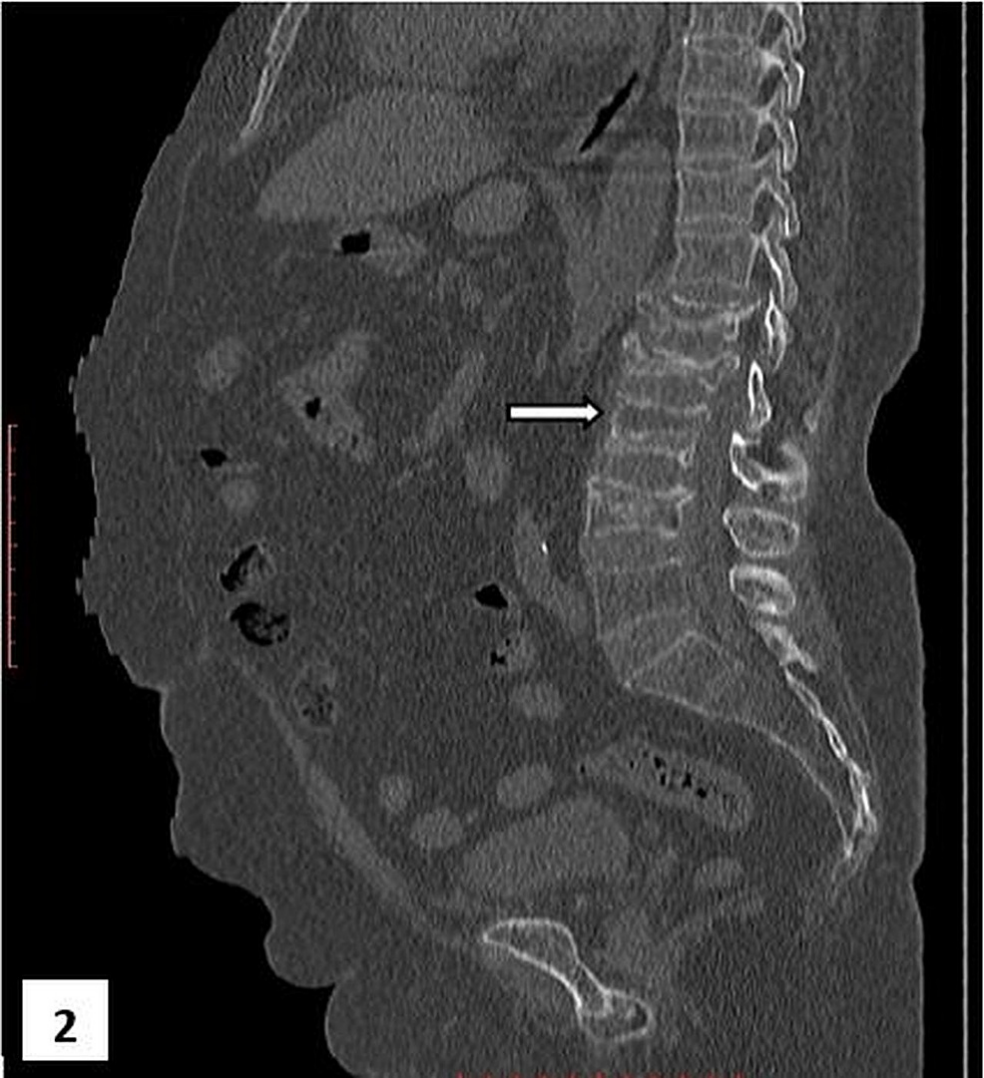
Computed tomography (saggital view) of the lumbosacral spine in the bone window The collapse of multiple vertebrae, a marked reduction in the vertebral body height, and a linear intraosseous cleft in L2 (arrow) are shown. L: lumbar.

A lateral radiograph of the lumbosacral spine showed compression wedge fractures at the L1, L2, L3, and L4 levels (Figure 1B). Computed tomography of the spine revealed the collapse of multiple vertebrae and a marked reduction in the vertebral body height accompanied by a linear intraosseous cleft in the L2 intervertebral disc (Figure 2).

**Figure 3:**
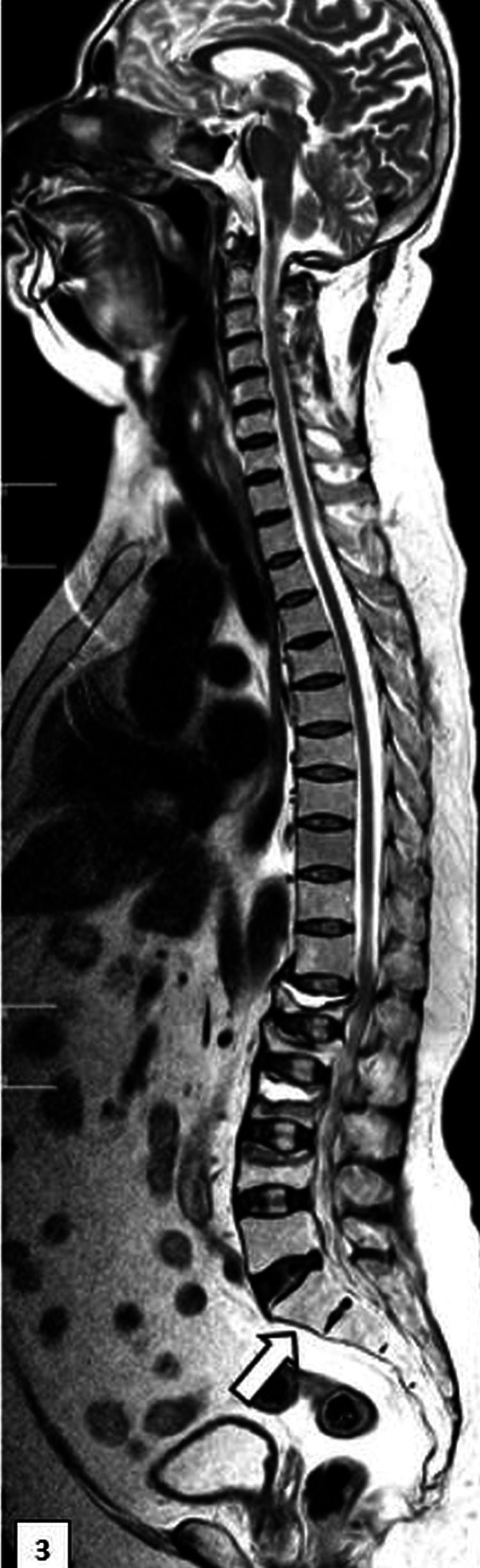
Magnetic resonance image of the whole spine Loss of normal curvature with sacralization of the lumbar vertebrae is shown.

**Figure 4:**
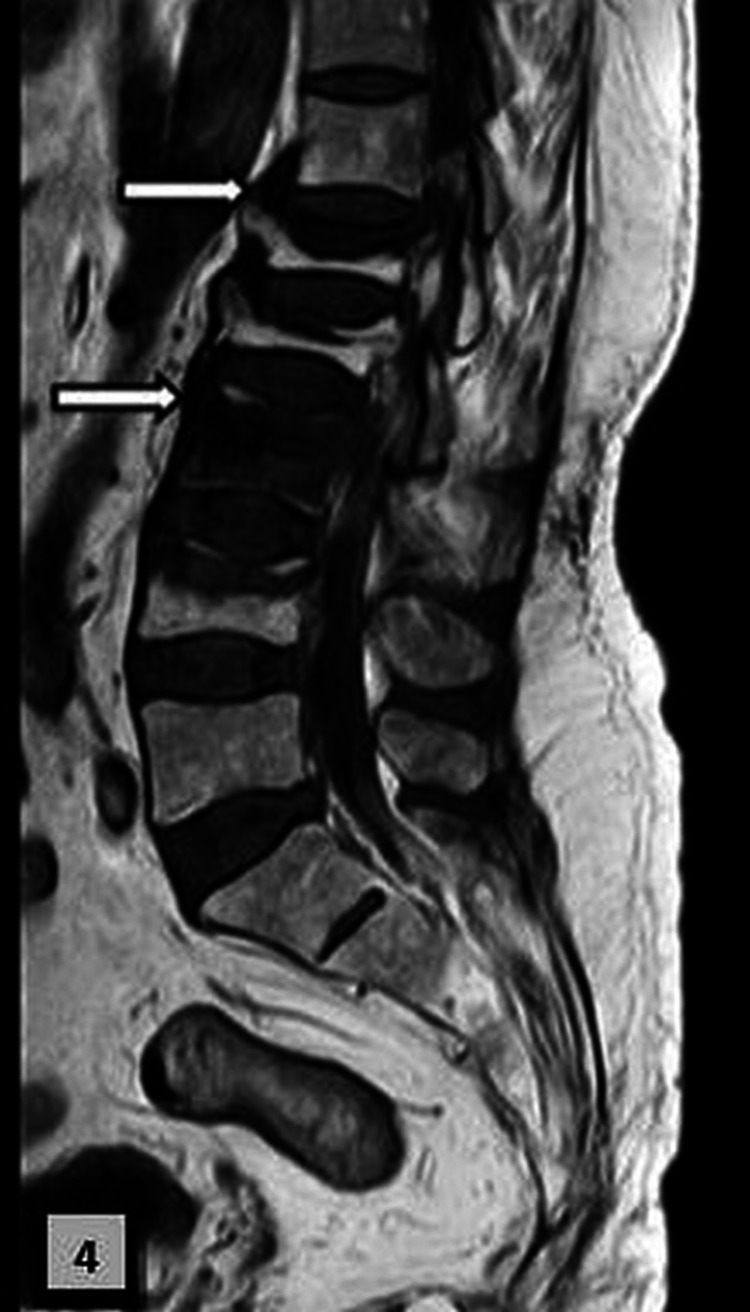
Magnetic resonance image of the lumbosacral spine (sagittal T1-weighted image) Hypointense signals below the superior endplates of L1 and L3 (arrows) are shown. L: lumbar.

**Figure 5:**
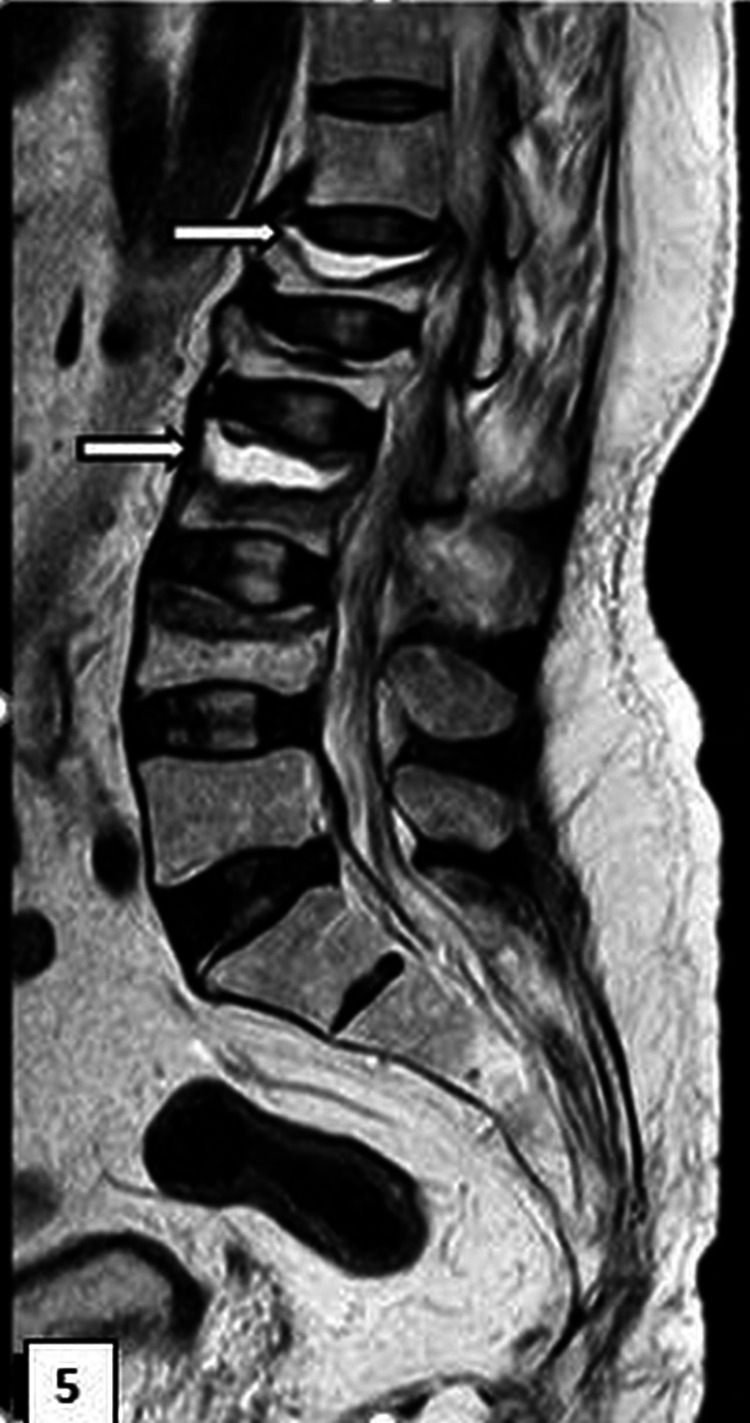
Magnetic resonance image of the lumbosacral spine (sagittal T2-weighted image) A fluid cleft (arrow) below the superior endplates of L1 and L3, indicative of Kummell’s disease, is shown. L: lumbar.

An extensive workup was done to exclude malignancy and infection. Magnetic resonance imaging revealed fluid clefts below the superior endplates of the D12 and L2 intervertebral discs (Figures 3-5).

**Figure 6:**
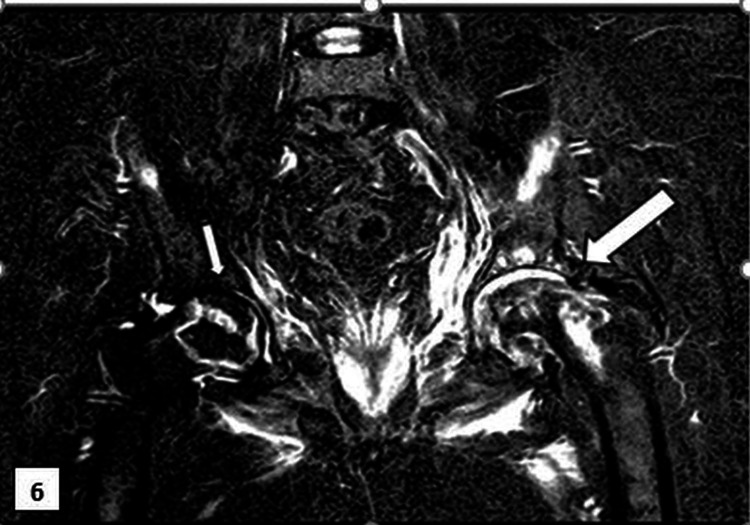
Magnetic resonance short-TI inversion recovery image (coronal image) Both hip joints reveal hyperintensities in the femoral heads, which are more severe on the left, and femoral head collapse accompanied by increased acetabular femoral joint space, suggestive of grade III avascular necrosis on the left side (big arrow) and grade I avascular necrosis (small arrow) on the right side. A mild amount of fluid is noted in both joints.

Coronal images of the hip joints obtained using magnetic resonance short-TI inversion recovery revealed hyperintensities in both femoral heads. More severity was noticed on the left side hip, with collapsed left femoral head and an increased acetabular femoral joint space. The findings were suggestive of grade III AVN on the left side and grade I AVN on the right side (Figure 6).

Kyphoplasty was advised, but the patient did not consent. Therefore, the nonsurgical treatment was bed rest, wearing a brace, lumbar traction, analgesics, and medication to prevent osteoporosis.

## Discussion

KD is a delayed post-traumatic avascular osteonecrosis of the vertebral body secondary to a vertebral compression fracture; it affects the vertebra between T8 and L4, and it is most frequently (60%) found between T11 and L1 [2]. In rare cases, multiple spinal metamers are involved [3]. Although KD is a frequent cause of post-traumatic vertebral compression fracture that is characterized as AVN of the vertebral body [4], it remains relatively rare and unknown to many. As a result, it is likely that some doctors will be involved in its treatment and prognosis at some point in their careers. Knowledge of its characteristics is important because it will simplify administering care, making an early diagnosis, and reducing unnecessary procedures.

The clinical stages of KD have been divided by Steele into five stages [5] (Table 1). (1) The initial injury stage with varied trauma, but the X-ray is negative; (2) second post-traumatic stage during which patients are mostly asymptomatic and have no restrictions to their activity; (3) third latent interval stage during which patients are relatively well but can later progress to a disability, not debilitation; (4) fourth recrudescent stage during which patients often complain of localized pain because pain often progresses as peripheral root pain; and (5) final terminal stage during which kyphosis forms, which can be associated with or without spinal cord compression [5].

**Table 1:** Clinical stages of Kummels disease [5]

Clinical stages of Kummels Disease [5]	
Stage 1	Injury stage with varied trauma, but the X-ray is negative
Stage 2	The post-traumatic stage during which patients are mostly asymptomatic and have no restrictions to their activity
Stage 3	Latent interval stage during which patients are relatively well but can later progress to a disability, not debilitation
Stage 4	The recrudescent stage during which patients often complain of localized pain because pain often progresses as peripheral root pain
Stage 5	The terminal stage during which kyphosis forms, which can be associated with or without spinal cord compression

The mechanism that causes delayed posttraumatic osteonecrosis of the vertebral body is unclear, but several pathophysiological mechanisms have been proposed. These mechanisms include atrophic non-union microfracture, fatigue fracture, pseudoarthrosis, and AVN secondary to vertebral body compression [6]. Furthermore, the nonspecific symptomology, spontaneous resolution of initial pain, difficulty in recalling minor trauma, and absence of specific diagnostic tests render KD a challenge to diagnose. However, interestingly, KD is often associated with osteoporosis or is found in patients predisposed to osteoporosis. Although KD can occur several months or years after an initial spinal injury, it is distinguished from a typical osteoporotic compression fracture in that its development is delayed.

After a diagnosis of vertebral body compression, a detailed general medical evaluation must be obtained, given that vertebral body compression can also occur in other conditions such as neoplasms, infection, osteoporosis, and predisposing factors to osteonecrosis. A careful review of images and exclusion of other common causes (including infection and malignant neoplasia) of vertebral body compression by using radiology is essential to ensure that the correct diagnosis is made. The best use of radiology for diagnosing KD is serial imaging, which shows the structure of the vertebral body before and after the vertebral collapse. On radiographs, an intravertebral vacuum cleft (IVC) filled with gas or fluid at the time of imaging, referred to as the “Kummell sign,” is described as pathognomic of KD [7]. In the case described herein, magnetic resonance imaging showed fluid clefts below the superior endplates of L1 and L3, and magnetic resonance short-TI inversion recovery imaging confirmed delayed posttraumatic vertebral collapse. In this patient, an intervertebral fluid cleft in the L2 intervertebral disc and secondary kyphosis could have resulted in the development of KD.

One of the most characteristic features of KD is delayed healing or nonhealing due to osteonecrosis of fractured vertebrae and loss of bone tissue that leads to the collapse of the vertebral body after a delay of months or years. Osteonecrosis can be caused by anatomical or mechanical damage to the blood vessel supplying blood to the vertebral body. Osteonecrosis can easily occur in the anterior 1/3 of the vertebral body, as the site is vulnerable to ischemic injury. Libicher et al. analyzed the histological and radiological findings of 180 vertebral compression fractures and confirmed intravertebral vacuum cleft as an accurate sign of osteonecrosis [8]. Although rare, delayed vertebral osteonecrosis with osteoporotic compression fractures should be considered for advanced radiological examinations from the initial stage of fracture.

The treatment and prognosis of KD rely on pain severity, degree of kyphotic deformity, and neurological deficits requiring surgical intervention. Early studies have focused on conservative treatment, employing medical pain management, bed rest, and bracing. However, recent studies support surgical procedures, which provide the advantage of earlier patient ambulation and correction of the kyphotic deformity. In patients in whom surgery fails or the extent of kyphotic deformity is extreme, minimally invasive surgical procedures such as vertebroplasty or kyphoplasty are used to treat spinal compression fracture, thus relieving pain, stabilizing the vertebra, and restoring it to its normal height [9]. Kyphoplasty and vertebroplasty are efficient in achieving immediate pain relief and improving mobility via stabilization of the fracture cleft. In other cases, patients may need corpectomy or disc excision to address complications resulting from neurologic compression [10]. Unfortunately, our patient refused to undergo surgical treatment, so the next best option was conservative management.

## Conclusions

Back pain is a common symptom observed in outpatient clinics. This case report and radiological images highlight the characteristic findings of KD for clinicians. Possessing the knowledge of KD and performing imaging at sequential intervals can help clinicians identify the cause and determine the proper treatment. Further, essential knowledge and intervention are essential for early identification of KD and prevention of long-term debilitating sequelae associated with KD.
